# Seminal fluid‐mediated fitness effects in the simultaneously hermaphroditic flatworm *Macrostomum lignano*

**DOI:** 10.1002/ece3.5825

**Published:** 2019-11-18

**Authors:** Michael Weber, Athina Giannakara, Steven A. Ramm

**Affiliations:** ^1^ Department of Evolutionary Biology Bielefeld University Bielefeld Germany

**Keywords:** male–male competition, multiple mating, seminal fluid, sexual selection, sperm competition, sperm precedence

## Abstract

As a class, seminal fluid proteins are expected to exert strong effects on mating partners due to the selection pressures of sperm competition and sexual conflict. But because of the complexity of this secretion, linking specific proteins to downstream effects on own fitness—via manipulating the reproductive behavior, physiology, and ultimately the sperm utilization of mating partners—is not straightforward. Here, we adopted a systematic gene knockdown approach to screen for seminal fluid‐mediated fitness effects in the simultaneously hermaphroditic flatworm *Macrostomum lignano*. We focused on 18 transcripts in *M. lignano* seminal fluid, testing how their RNA interference‐induced knockdown impacted on three aspects of donor (male) reproductive success: (a) fertility (offspring production of the partner); (b) defensive sperm competitive ability, *P*_1_; and (c) offensive sperm competitive ability, *P*_2_. In general, the knockdown of most individual transcripts appeared to have only a minor impact on male reproductive success, though we found evidence that the knockdown of up to five different transcripts impacted on fertility; the knockdown of two other transcripts resulted in reduced *P*_2_; and knockdown of a further transcript actually increased *P*_2_. We thus identify a number of candidate seminal fluid transcripts that appear to modulate offspring production and sperm competitiveness in *M. lignano*. That only a minority of transcripts exhibit such a pattern likely reflects both the difficulty of accurately estimating sperm competitiveness and the functional redundancy of seminal fluid.

## Graphical abstract

We identify a number of candidate seminal fluid transcripts that appear to modulate offspring production and sperm competitiveness in the simultaneously hermaphroditic flatworm *Macrostomum lignano*. That only a minority of transcripts exhibit such a pattern likely reflects both the difficulty of accurately estimating sperm competitiveness and the functional redundancy of seminal fluid.

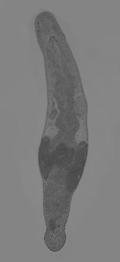

## 1 INTRODUCTION

In animals with internal fertilization, not only sperm but also a complex mixture of additional male‐derived substances—known collectively as seminal fluid—is transferred to the female reproductive tract during insemination (Poiani, 2006). The seminal fluid proteins (SFPs; also referred to as accessory gland proteins (ACPs) or male accessory gland (MAG) proteins in other species) found in this secretion play a crucial role in reproduction and can affect the behavior and/or physiology of a partner who receives them, thereby influencing both male and female reproductive success (reviewed in: Avila, Sirot, Laflamme, Rubinstein, & Wolfner, 2011; Chapman, 2001; Hopkins, Sepil, & Wigby, 2017; Sirot, Wong, Chapman, & Wolfner, 2015). From a male perspective, SFPs likely affect both the ability of an individual's ejaculate to resist displacement by a second male's ejaculate (defensive sperm competitive ability, usually measured as paternity share as the first to mate, *P*_1_) and/or to induce displacement of a previous male's ejaculate (offensive sperm competitive ability, measured as paternity share as the second to mate, *P*_2_; Boorman & Parker, 1976; Clark, Begun, & Prout, 1999; Parker, 1970).

Seminal fluid could mediate sperm competitive ability through multiple mechanisms. Some SFPs play important roles in nourishing and activating the sperm themselves (Mann & Lutwak‐Mann, 1982). Other SFPs influence—either independently of, or in association with, sperm (Peng et al., 2005)—subsequent female physiology and behavior in various ways (reviewed in: Avila et al., 2011; Hopkins et al., 2017; Poiani, 2006; Sirot et al., 2015). A frequent effect of SFPs reported among female insects is, for example, an increase in egg production, ovulation, and/or egg‐laying rates (Avila et al., 2011; Gillott, 2003; Poiani, 2006; Ram & Wolfner, 2007a). Other SFPs exert their effects via modulating sperm storage. For example, in *Drosophila*, Acp36DE is required for efficient storage of sperm (Neubaum & Wolfner, 1999; Qazi, 2003) and because females mated to males lacking Acp36DE store fewer sperm than mates of normal males, Acp36DE‐null males achieve significantly fewer fertilizations as second males following double matings (Chapman, Neubaum, Wolfner, & Partridge, 2000). Also in *Drosophila melanogaster*, ejaculate receipt changes female behavior such that mated females actively reject courting males; the SFP sex peptide plays a central role in inducing this change (Chapman et al., 2003; Häsemeyer, Yapici, Heberlein, & Dickson, 2009; Liu & Kubli, 2003; Ram & Wolfner, 2009; Yang et al., 2009; Yapici, Kim, Ribeiro, & Dickson, 2008).

Because SFPs and other molecules in seminal fluid can profoundly affect the behavior and/or physiology of the female who receives them, ultimately influencing male reproductive success through differential sperm competitive ability, SFPs are important targets of sexual selection (Cameron, Day, & Rowe, 2007; Chapman, 2001; Hodgson & Hosken, 2006; Poiani, 2006; Ram & Wolfner, 2007a) and mediators of sexual conflict (Arnqvist & Rowe, 2005; Chapman et al., 1995; Sirot et al., 2015). This likely also explains their often rapid adaptive evolution (Andrés et al., 2006; Clark, Aguade, Prout, Harshman, & Langley, 1995; Clark, Aagaard, & Swanson, 2006; Clark & Swanson, 2005; Haerty et al., 2007; Ramm, Oliver, Ponting, Stockley, & Emes, 2008; Swanson, Clark, Waldrip‐Dail, Wolfner, & Aquadro, 2001). One consequence of this is that it can be difficult to identify SFP homology between species and there are likely to be many lineage‐specific functions (Clark et al., 2006; Clark & Swanson, 2005; Haerty et al., 2007; Swanson et al., 2001; Swanson & Vacquier, 2002), although the major protein classes found in SFPs are conserved between taxa as distantly related as insects and mammals (Mueller, Ripoll, Aquadro, & Wolfner, 2004).

In order to functionally characterize seminal fluid, previous studies have often sought either to artificially inject and monitor female responses to specific proteins (e.g., Kingan, Thomas‐Laemont, & Raina, 1993; Koene et al., 2010; Nakadera et al., 2014; Shutt, Stables, Aboagye‐Antwi, Moran, & Tripet, 2010; Takami, Sasabe, Nagata, & Sota, 2008; Wigby & Chapman, 2005; Yamane, Miyatake, & Kimura, 2008) or else to genetically modify males such that naturally transferred ejaculates are missing specific SFPs (e.g., Chapman et al., 2000; Herndon & Wolfner, 1995; Liu & Kubli, 2003; Mueller, Linklater, Ram, Chapman, & Wolfner, 2008; Neubaum & Wolfner, 1999; Peng et al., 2005; Qazi, 2003; Wong et al., 2008). Unfortunately, it is not always possible to collect or manipulate whole ejaculates, or to create transgenic animals missing specific SFPs, especially in nonmodel organisms. These constraints can, however, largely be overcome with the use of RNA interference (RNAi) technology to induce targeted suppression of gene expression. RNAi has enabled researchers to identify the mechanisms underlying a range of SFP‐mediated physiological traits, including egg production, sexual receptivity to remating and sperm storage in, for example, *Drosophila* (Chapman et al., 2003; Ram & Wolfner, 2007b; Sirot et al., 2009; Sitnik, Gligorov, Maeda, Karch, & Wolfner, 2016), crickets (Marshall et al., 2009), and beetles (Xu, Baulding, & Palli, 2013). *Drosophila melanogaster* females mated to sex peptide knockdown males were significantly more receptive and laid and ovulated significantly fewer eggs than did mates of control males (Chapman et al., 2003). Females mated to CG10586 knockdown males showed a lower level of egg laying and higher rates of sexual receptivity to subsequent males (LaFlamme, Ravi Ram, & Wolfner, 2012). In the cricket *Allonemobius socius*, EJAC‐SP knockdown males had a reduced ability to induce a female to lay eggs (Marshall et al., 2009), and in the red flour beetle, *Tribolium castaneum*, knockdown of an angiotensin‐converting enzyme in seminal fluid reduced egg production by mated females (Xu et al., 2013).

**Figure 1:**
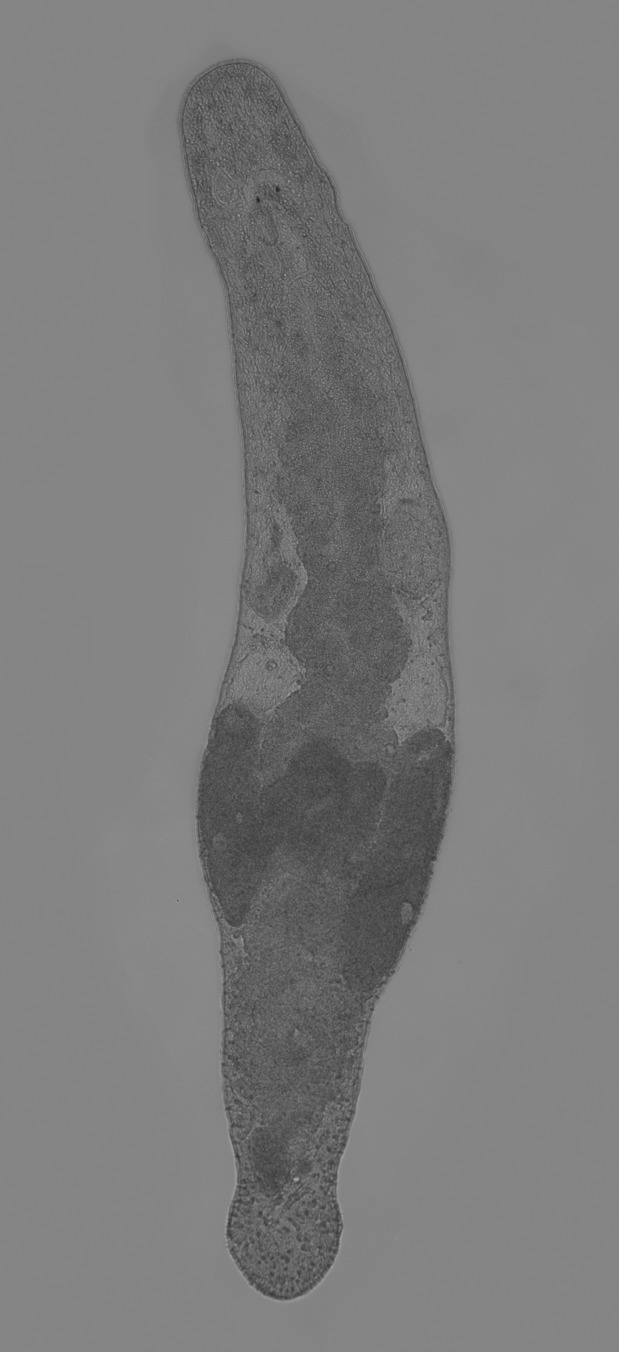
Simultaneously hermaphroditic flatworm *Macrostomum lignano*

In this study, we sought to use RNAi to test for SFP‐mediated effects on reproduction in the simultaneously hermaphroditic flatworm *Macrostomum lignano* (Figure 1). This species is a promising model organism in which to extend the taxonomic range of seminal fluid studies both because it is a simultaneous hermaphrodite—which might create unique targets of seminal fluid action (Charnov, 1979; Schärer, Janicke, & Ramm, 2015; Schärer & Ramm, 2016) such as change of partners resource allocation to the male and female sex functions or the amount of sperm a recipient transfers in its next mating (Nakadera et al., 2014)—and due to useful biological features such as its transparency, which enables the in vivo observation of relevant reproductive traits such as gonad size or the quantification of received sperm (Marie‐Orleach, Janicke, Vizoso, David, & Schärer, 2016; Marie‐Orleach, Janicke, Vizoso, Eichmann, & Schärer, 2014; Schärer & Ladurner, 2003).

Although the complement of seminal fluid proteins in *M. lignano* has only just been characterized (Weber et al., 2018), there are already some indications for potential effects of SFPs. Specifically, previous work indicates that individuals mated to virgin partners (which presumably transfer bigger ejaculates containing more sperm and/or SFPs than do recently mated “SFP‐depleted” individuals due to having larger seminal vesicles and more stored seminal fluid than recently mated individuals) exhibited a lower frequency of the so‐called suck behavior (Marie‐Orleach, Janicke, & Schärer, 2013). A similar reduction in postmating responses was shown for *Drosophila* females which were mated to SFP‐depleted males (Linklater, Wertheim, Wigby, & Chapman, 2007; Sirot et al., 2009). The postmating “suck” response of worms to ejaculate receipt—during which the individual places its pharynx over its own female genital opening and appears to attempt to suck out its contents—is proposed to function to remove either the sperm or SFPs previously received during copulation (Schärer, Joss, & Sandner, 2004; Scharer, Littlewood, Waeschenbach, Yoshida, & Vizoso, 2011; Vizoso, Rieger, & Schärer, 2010). Another recent study showed that individuals with a higher testis investment sire a higher proportion of offspring under sperm competition than do individuals with lower testis investment (Vellnow, Marie‐Orleach, Zadesenets, & Schärer, 2018). Because increased testis investment in high mating environments is accompanied also by an increase in SFP transcript expression (Ramm et al., 2019), this effect could be partially mediated through seminal fluid though of course it may be largely explained by differences in sperm numbers (Sekii et al., 2013; Vellnow et al., 2018) in this competitive scenario. We here aimed to provide a more direct test for seminal fluid‐mediated fitness effects in *M. lignano*, by screening 18 putative SFPs, identified by in situ hybridization screening for prostate‐limited expression (Weber et al., 2018), for the effects of RNAi‐induced knockdown on both fertility (measured as the offspring production of mating partners) and defensive and offensive aspects of sperm competitive ability.

## 2 METHODS

### 2.1 Study organism and experimental subjects

The free‐living flatworm *M. lignano* is an outcrossing simultaneous hermaphrodite found in the Northern Adriatic Sea and Eastern Mediterranean (Ladurner, Schärer, Salvenmoser, & Rieger, 2005; Zadesenets et al., 2016). As adults, the worms reach ca. 1.5 mm in body length and the paired male and female gonads lay along the body axis on either side of a central gut. The male and female genital organs are located in the posterior part of the worms, and the former includes the prostate gland cells where seminal fluid is produced (Hyman, 1951; Weber et al., 2018). The worms are kept in cultures in glass petri dishes filled with artificial seawater (ASW, 32‰) or nutrient‐enriched artificial seawater (Guillard's f/2 medium; Guillard & Ryther, 1962) and fed with diatoms (*Nitzschia curvilineata*). They are kept under standard conditions on a 14:10 light:dark cycle at 60% relative humidity and a constant temperature of 20°C. All the animals used in this experiment as knockdown/control donors and as recipients (see below) belonged to the highly inbred DV1 line (Janicke et al., 2013) that was previously used to identify putative seminal fluid candidates (Weber et al., 2018).

In this study, we needed to assign paternity to offspring of competing ejaculate donors (i.e., what would be competing males in separate‐sexed animals). To do so, we used as sperm competitors individuals from an outbred transgenic BAS1 line of *M. lignano* that expresses GFP ubiquitously (Marie‐Orleach et al., 2016; Vellnow et al., 2018), such that in double‐mating trials, the resulting offspring could be unambiguously assigned as being sired by either the DV1 (GFP^−^) or BAS1 (GFP^+^) worm (see also Janicke et al., 2013; Marie‐Orleach et al., 2014, which employed a GFP‐expressing inbred line [HUB1] for the same purpose). Offspring production, mating rate, and morphology were previously found not to differ significantly between GFP^+^ and GFP^−^ individuals (Marie‐Orleach et al., 2014).

To reduce among‐individual variation due to age, we used closely age‐matched individuals in all experiments. For this, we transferred well‐fed adult individuals into glass petri dishes with ASW and ad libitum algae, and allowed individuals to lay eggs for 2 days before removing them again, ensuring the resulting hatchlings that we allocated as experimental subjects did not differ in age by more than 2 days. All animals used in the experiment were, at the day of their first mating trial, 60 ± 1 days old.

### 2.2 Selection of candidates and experimental design

Our study represents a “naïve” screen for seminal fluid‐mediated fitness effects in a species for which we have no prior functional information on specific SFPs. There are also no sequence similarities to already known SFPs or other substances in better investigated species. Therefore, to prioritize SFP candidates for screening, we first selected them based on their phenotypic plasticity in gene expression investigated in a recent RNA‐Seq study (Ramm et al., 2019). We selected transcripts with prostate‐limited expression that exhibited markedly higher expression in an environment with a high sperm competition level (compared to isolated worms), on the basis that these might be more likely to play important roles in sperm competition. Second, the candidate list was refined based on the confirmed efficacy of RNAi knockdown verified by performing whole‐mount in situ hybridization (ISH) in preliminary experiments (data not shown). We thus investigated RNAi knockdown effects for Mlig‐pro4, 5, 7, 8, 10, 11, 13, 23, 28, 34, 35, 38, 46, 49, 54, 60, 63, and 69 (see Weber et al., 2018 for details about nomenclature, but note that lower numbers represent transcripts with higher levels of overall expression in octets). All the SFPs investigated in this study exhibit bioinformatic evidence of being secreted and therefore of being transferred (Weber et al., 2018), though direct experimental confirmation of this is currently lacking.

For each RNAi/control treatment, we then conducted three separate assays with three separate batches of donor, competitor, and recipient worms, to measure (a) fertility, (b) defensive sperm competitive ability (*P*_1_), and (c) offensive sperm competitive ability (*P*_2_), respectively. Initially, worms in all three assays were treated identically, as described in the following three subsections. After the assays, the proper efficiency of transcript knockdowns in the RNAi donor worms was confirmed by ISH (see Figure [Link], [Link]). ISH was performed according to Lengerer et al. (2014). We could confirm for all 18 individual knockdowns a drastic decrease in expression (Figure [Link], [Link]) but ISH does not permit us to quantify this precisely. We therefore cannot rule out the possibility that still a small amount of the respective SFP was produced and transferred.

### 2.3 Raising conditions

Six to eight days posthatching, a batch of same‐age hatchlings was collected and distributed individually in wells of 24‐well tissue culture plates (TPP, Trasadingen, Switzerland) each filled with 1 ml of ASW and fed ad libitum with algae. Individuals were fed once per week with new algae. Recipient worms remained in the 24‐well tissue culture plates until they were used for the experiment (day 60). Donor worms (GFP^+^ and GFP^−^) remained in the 24‐well tissue culture plates until they underwent tail amputation (day 50, see below).

### 2.4 RNA interference

RNAi was performed as previously described (Kuales et al., 2011). Briefly, for each of the 18 seminal fluid candidates, a double‐stranded RNA (dsRNA) probe was generated by an in vitro transcription system using primer pairs with T7 and SP6 promoter regions (T7 and SP6 Ribomax™ large‐scale RNA kit, Promega; see Table [Link], [Link] for a list of primers). In addition, we used two control treatments (to control for dsRNA treatment effects) with either dsRNA for firefly luciferase (Arbore et al., 2015; Pfister et al., 2008; Sekii, Salvenmoser, Mulder, Scharer, & Ladurner, 2009) or water; control individuals were otherwise treated identically to the knockdown individuals.

Taking advantage of the regenerative capacity of *M. lignano* (Egger, Ladurner, Nimeth, Gschwentner, & Rieger, 2006), before beginning the RNAi (or control) treatment, adult virgin GFP^−^ donor animals were tail‐amputated between the antrum and ovaries to remove (a) the antrum, with all potential previously received ejaculate in it; (b) the seminal vesicle, with potential (own) stored sperm; as well as (c) the SFP‐producing prostate gland cells. This procedure ensured that seminal fluid production was “reset” prior to the RNAi/control treatment, which would have tended to equalize the amount and age of stored sperm and seminal fluid reserves and further meant that donor individuals also contained no received sperm or seminal fluid at the beginning of the mating trials (see below). After amputation, individuals were treated with dsRNA during the entire regeneration process and kept individually in a well of a 60‐well microtest plate (Greiner Bio‐One™ 60‐well HLA Terasaki Plates). Each worm was placed in 10 µl dsRNA solution (~25 ng/µl dsRNA for the specific transcript in ASW‐algae mix). Throughout the whole experiment, animals were fed ad libitum with algae and were maintained under standard culture conditions. On days 2, 4, 6, 8, and 10 postamputation, 2 µl of dsRNA solution was added to each well, and on days 3, 5, 7, and 9, the worms were transferred to a new well containing 10 µl of new dsRNA solution to ensure a constant exposure to dsRNA. The first mating trial was conducted on day 11 postamputation, which is sufficient for complete regeneration (Egger et al., 2006; Lengerer et al., 2018).

The GFP^+^ donor worms used in the experiment as sperm competitors to the experimental subjects were also 60 ± 1‐day‐old adult virgin worms, and were also tail‐amputated on the same day as the knockdown/control worms. Thereafter, they were also each kept individually in one well of a 60‐well microtest plate in 10 µl ASW with ad libitum algae. GFP^+^ worms were transferred to a new well containing 10 µl ASW with ad libitum algae once on day 5 postamputation.

### 2.5 Mating trials for noncompetitive (fertility) and competitive (*P*_1_ and *P*_2_) assays

Mating trials were conducted on days 11, 12, and 13 postamputation. Each treatment/control donor worm was paired with a new recipient worm in a 60‐well microtest plate in 8 µl ASW on each of these three consecutive days, with the recipient treated differently according to the type of assay (fertility, *P*_1_ or *P*_2_—see below). The mating pairs stayed together throughout the whole period (6 or 3 hr, depending on the type of assay); *Macrostomum* shows an average mating rate of about 6–15 copulations per hour (Janicke & Schärer, 2009; Marie‐Orleach et al., 2013; Schärer et al., 2004) and so we also expected multiple matings to have occurred among all mating pairs in all three assays we conducted. This design involving all donors being paired with three recipients was adopted to reduce measurement error, because of the expected relatively low offspring production per recipient. Irrespective of the assay type, to be able to distinguish individual worms under normal light during the mating trial, we colored all the recipient worms 24 hr beforehand using the food coloring dye Grand Bleu [E131 and E151] (Les Artistes—Paris), diluted to a concentration of 0.25 mg/ml in ASW. Such a 24‐hr exposure enables us to easily distinguish colored from noncolored worms and has previously been shown not to affect the mating rate (Marie‐Orleach et al., 2013). Between the three mating trials, the donor worms were kept overnight in new dsRNA solution or the respective control treatment. Recipients (used only once) were kept after their mating trial in 60‐well plates in ASW with ad libitum algae, and offspring production was monitored (the number of unhatched offspring was negligible and therefore ignored). They were transferred to a new well every second day until day 11 (6 wells in total), where they remained until day 21 (after which no further offspring were detected in any of the three assays).

### 2.6 Fertility assay

On each of the three consecutive mating trial days, focal worms (knockdown or control) were paired together for 6 hr with a randomly selected virgin recipient worm. After the 6‐hr mating period each day, the donors and recipients were processed as described above. Each treatment group began with 48 donor worms at the start of the RNAi treatment, but some individuals were lost either during the RNAi treatment or the mating trials due to pipetting mistakes, incomplete regeneration, sickness, or death. The final sample size for each treatment group therefore ranged from 44 to 48 donors (and correspondingly from 127 to 142 recipients); in total, we scored the paternity of 13,490 hatchlings.

### 2.7 Sperm competitive ability assays (*P*_1_ and *P*_2_)

To estimate defensive sperm competitive ability (*P*_1_), either knockdown or control worms were mated for three hours with a randomly selected virgin recipient worm. After three hours, the donor worms were removed and (on days 1 and 2) put back into their respective treatments as described above. One hour after removing the knockdown/control worm, a randomly selected GFP^+^ sperm competitor worm was added to the well containing the already‐mated recipient worm, and the pair were allowed to mate for a further 3 hr. Again, each donor worm (and its same competitor) was mated consecutively with three recipient worms on three consecutive days, and recipients were subsequently processed as described above. The resulting offspring were counted and categorized to either GFP^−^ (sired by first knockdown or control donor) or GFP^+^ (sired by the competitor donor) until day 21, based on expression of GFP assessed at age 7–10 days using a Nikon SMZ‐18 stereomicroscope with a C‐HGFI Intensilight fluorescence light source and GFP filter cube (Nikon GmbH, Düsseldorf, Germany). Each treatment group started with 24 donor worms at the beginning of the RNAi treatment. With some loss as described for the fertility assay, the final realized sample sizes for each treatment group ranged from 18 to 23 donors (and correspondingly from 34 to 52 recipients), and in total, we scored the paternity of 3,139 hatchlings.

To estimate offensive sperm competitive ability (*P*_2_), the sperm competition assay was carried out exactly like the *P*_1_ assay, except that the GFP^+^ worm was paired with the recipient first and the knockdown/control worm second. The final sample size for each treatment group ranged from 18 to 23 donors (and correspondingly from 34 to 50 recipients), and in total, we scored the paternity of 3,053 hatchlings.

### 2.8 Statistical analysis

For the analysis of the fertility assay, the offspring number of all three recipients mated with the same donor worm was first summed up and then divided by the number of recipients (usually three, but sometimes less due to unsuccessful mating or death) to obtain an average offspring number per recipient. This procedure was followed because of a high number of recipients which did not produce any offspring. We therefore would have ended up with a zero‐inflated dataset which would have been more complicated to analyze and interpret. These “per capita” fertility measures for each donor were then compared across treatments using a linear model. For the analysis of the *P*_1_ and *P*_2_ assays, we compared the paternity share of knockdown and control individuals (GFP^−^) against the GFP competitor using a generalized linear model with a quasibinomial distribution, treatment as main factor, recipient order (1/2/3) as fixed effect, and donor ID as random effect (Engqvist, 2013). Because there was no significant difference between the two control treatments in any of the three assays (water vs. dsRNA for firefly luciferase; fertility: *t* = 1.191, *p* = .24; *P*_1_: *z* = 1.417, *p* = .16; *P*_2_: *z* = 0.694, *p* = .49), they were combined and treated as a single control group for subsequent analysis for simplicity. Analyses were conducted using the lme4 package for R (R version 3.1.3., 2015). All *p*‐values are reported both unadjusted and additionally adjusted for the false discovery rate (FDR) to control for multiple testing according to the method of Benjamini and Hochberg (1995).

## 3 RESULTS

### 3.1 SFP knockdown effects on fertility

**Figure 2:**
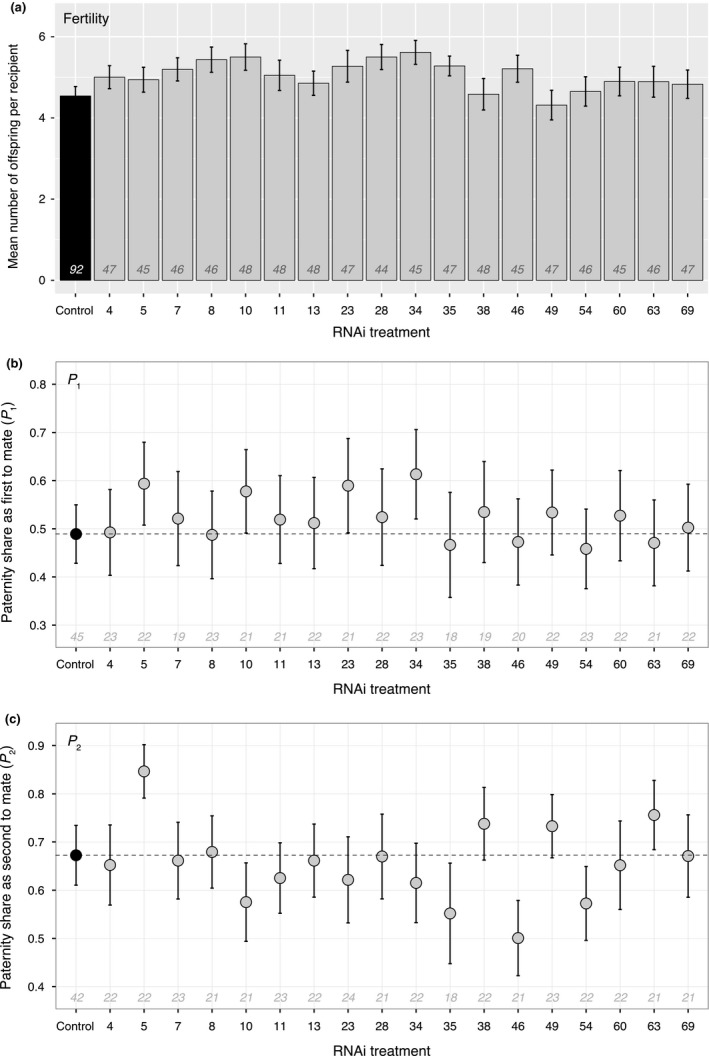
The effect of RNAi knockdown of 18 different seminal fluid transcripts (Mlig‐pro4, 5, 7, 8, 10, 11, 13, 23, 28, 34, 35, 38, 46, 49, 54, 60, 63, and 69) on offspring production in noncompetitive and paternity share in competitive fitness assays. The transcripts are labeled according to their Mlig‐pro[number] identifier assigned in Weber et al. (2018). Sample sizes (number of donors) are given in italics above each *x*‐axis. (a) Mean offspring produced per partner ± *SE* by knockdown (kept in dsRNA for the corresponding transcript) versus controls (either kept in ASW or in dsRNA for firefly luciferase) when individuals were mated with three virgin partners on three consecutive days. (b) Mean paternity share (*P*_1_) ±*SE* of knockdown versus control individuals mated with three virgin partners on three consecutive days when the RNAi/control worm mated first and the GFP‐expressing competitor second. (c) Mean paternity share (*P*_2_) ±*SE* of knockdown versus control individuals mated with three virgin partners on three consecutive days when the GFP‐expressing competitor mated first and the RNAi/control worm second

When we compared recipient worms mated with SFP knockdown donors to those mated to the control donors, five knockdowns (Mlig‐pro8, 10, 28, 34, and 35) showed a significant increase in offspring production before FDR correction, as measured by total offspring production per recipient worms in the first 21 days following the trial. However, none of these individual knockdowns had a significant impact on offspring production after FDR correction to control for multiple testing (all *p*_adjusted_ ≥ .1, Figure 2a; for full statistical details for each knockdown, see Table 1).

**Table 1:** Descriptive statistics and tests for treatment effects on fertility and sperm competitive ability following RNAi knockdown of 18 seminal fluid transcripts. (a) Fertility assay. (b) Competitive assay with treatment individuals as first mating partner (*P*_1_). (c) Competitive assay with treatment individuals as second mating partner (*P*_2_)

Treatment	Fertility						*P*_1_						*P*_2_					
	Mean offspring/recipient	Std. error	*df*	*t*‐Value	*p*‐Value (unadjusted)	*p*‐Value (adjusted)	Mean paternity share (*P*_1_)	Std. error	*df*	*z*‐Value	*p*‐Value (unadjusted)	*p*‐Value (adjusted)	Mean paternity share (*P*_2_)	Std. error	*df*	*z*‐Value	*p*‐Value (unadjusted)	*p*‐Value (adjusted)
Control	4.54	0.23					0.49	0.06					0.67	0.06				
Mlig‐pro4	5.00	0.28	137	1.21	.23	.41	0.49	0.09	139	1.29	.20	.89	0.65	0.08	136	0.25	.81	.90
Mlig‐pro5	4.94	0.31	135	1.01	.31	.51	0.59	0.09	129	1.73	.09	.89	0.85	0.06	137	2.18	.03	.24
Mlig‐pro7	5.20	0.29	136	1.70	.09	.23	0.52	0.10	125	0.65	.51	.89	0.66	0.08	136	0.37	.72	.90
Mlig‐pro8	5.43	0.31	136	2.26	.025	.11	0.49	0.09	133	−0.34	.73	.89	0.68	0.08	133	0.67	.50	.90
Mlig‐pro10	5.50	0.33	138	2.41	.017	.10	0.58	0.09	127	0.50	.62	.89	0.58	0.08	130	−1.30	.20	.58
Mlig‐pro11	5.05	0.37	138	1.21	.23	.41	0.52	0.09	130	0.29	.77	.89	0.63	0.07	137	−0.52	.61	.90
Mlig‐pro13	4.85	0.30	138	0.81	.42	.54	0.51	0.09	134	0.82	.41	.89	0.66	0.08	137	0.20	.84	.90
Mlig‐pro23	5.27	0.39	137	1.71	.09	.23	0.59	0.10	129	1.57	.12	.89	0.62	0.09	127	0.19	.85	.90
Mlig‐pro28	5.50	0.31	134	2.41	.017	.10	0.52	0.10	131	1.07	.29	.89	0.67	0.09	133	0.37	.72	.90
Mlig‐pro34	5.61	0.30	135	2.75	.007	.10	0.61	0.09	133	0.51	.61	.89	0.62	0.08	130	−1.22	.23	.58
Mlig‐pro35	5.28	0.24	137	2.00	.047	.17	0.47	0.11	126	−0.88	.38	.89	0.55	0.10	122	0.37	.71	.90
Mlig‐pro38	4.58	0.39	138	0.10	.92	.92	0.53	0.10	121	1.01	.31	.89	0.74	0.08	132	1.43	.16	.58
Mlig‐pro46	5.21	0.33	135	1.65	.10	.23	0.47	0.09	126	−0.13	.90	.89	0.50	0.08	135	−2.97	.0035	.06
Mlig‐pro49	4.32	0.37	137	−0.54	.59	.66	0.53	0.09	129	0.27	.79	.89	0.73	0.07	138	−0.32	.75	.90
Mlig‐pro54	4.65	0.36	136	0.27	.79	.84	0.46	0.08	137	−0.21	.83	.89	0.57	0.08	136	−2.08	.04	.24
Mlig‐pro60	4.90	0.35	135	0.86	.39	.54	0.53	0.09	131	0.69	.49	.89	0.65	0.09	135	−1.32	.19	.58
Mlig‐pro63	4.89	0.38	136	0.83	.41	.54	0.47	0.09	129	−0.46	.65	.89	0.76	0.07	130	0.81	.42	.90
Mlig‐pro69	4.83	0.35	137	0.70	.48	.58	0.50	0.09	128	−0.15	.88	.89	0.67	0.09	135	−0.01	.99	.99

### 3.2 SFP knockdown effects on defensive sperm competitive ability (*P*_1_)

The paternity share of SFP knockdown donor worms (GFP^−^) was compared to that of control donor worms (GFP^−^) when they mated as the first partner with a recipient worm who subsequently mated with an outbred sperm competitor (GFP^+^). None of the individual knockdowns appeared to impact strongly on this aspect of sperm competitive ability, with no significant differences in *P*_1_ between any of the treatments and the controls (all *p*_unadjusted_ ≥ .1, all *p*_adjusted_ = .89, Figure 2b; Table 1).

### 3.3 SFP knockdown effects on offensive sperm competitive ability (*P*_2_)

The paternity share of SFP knockdown donor worms (GFP^−^) was compared to that of control donor worms (GFP^−^) when they mated as the second partner with a recipient worm that had previously mated with an outbred sperm competitor (GFP^+^). By contrast to the *P*_1_ assays, but similar to the fertility assay, we here observed that a minority of knockdowns show some evidence of impacting sperm competitive ability (Figure 2c, Table 1). Mlig‐pro46 knockdown donors (*z* = −2.97, *p*_unadjusted_ = .0035, *p*_adjusted_ = .06) and Mlig‐pro54 knockdown donors (*z* = −2.08, *p*_unadjusted_ = .04, *p*_adjusted_ = .24) exhibited a reduced *P*_2_, whereas Mlig‐pro5 knockdown donors actually exhibited an increased *P*_2_ (*z* = 2.18, *p*_unadjusted_ = .03, *p*_adjusted_ = .24). Again, as for the fertility assay, these increases/decreases were significant before FDR correction but not after.

## 4 DISCUSSION

By choosing a subset of 18 putative seminal fluid transcripts with prostate‐limited expression (Weber et al., 2018) and subjecting these to RNAi knockdown followed by competitive and noncompetitive paternity assays, we sought to identify seminal fluid transcripts that modulate offspring production and sperm competitive ability in *M. lignano*. The majority of single knockdowns did not impact on these measures of male reproductive success, though our screen identified a number of candidates implicated in at least one aspect. Specifically, there was no difference in paternity share (*P*_1_) between knockdown and control individuals when the knockdown individuals were the first mating partners. By contrast, when the knockdown individuals were the second mating partners, two knockdowns (Mlig‐pro46 and Mlig‐pro54) exhibited a reduced *P*_2_, whereas another (Mlig‐pro5) exhibited an increased *P*_2_. Additionally, there was evidence for five knockdowns that donors had a higher fertility than controls, that is, their mating partners produced more offspring. Our study thus provides some evidence for seminal fluid‐mediated fitness effects, but we caution that all of these effects became nonsignificant after performing FDR correction to control for multiple testing. In part, this reflects the large‐scale nature of the screen we performed and despite the fact that we assigned paternity to almost 20,000 offspring in total, our power to detect differences in paternity outcomes for individual knockdowns and assays was somewhat limited, especially given the noisy nature of the outcomes we were measuring. Examining the effect sizes suggests that, if confirmed in subsequent studies now focusing on this smaller pool of candidates, some of the effects we have identified are actually quite marked, resulting in a 14%–19% change in fertility among the five candidates identified as impacting on this aspect of reproductive success, and 15%–35% for the three candidates affecting *P*_2_.

Our results suggest that Mlig‐pro46 and Mlig‐pro54 could play important functional roles in reproduction relevant to sperm competition outcomes, though this needs to be confirmed in a dedicated experiment and the precise mechanisms through which this could be mediated remain to be elucidated. We also do not know at this stage whether the knockdown of a specific transcript in our experiment impacted on the expression of other SFPs. Nevertheless, several possible mechanisms for the function of Mlig‐pro46 and Mlig‐pro54 would now be worth exploring. Based on evidence from other taxa, these proteins could, for example, be important for sperm storage (Chapman et al., 2000; Neubaum & Wolfner, 1999; Qazi, 2003), release of sperm from storage (Avila, Mattei, & Wolfner, 2015; Ram & Wolfner, 2007b), and sperm viability (den Boer, Baer, & Boomsma, 2010; den Boer, Baer, et al., 2009; den Boer, Boomsma, & Baer, 2009; Holman, 2009), and several other studies identified overall effects of SFPs on sperm competitive ability (e.g., Chapman et al., 2000; Clark et al., 1995; Fiumera, Dumont, & Clark, 2005; Fiumera, Dumont, & Clark, 2007; Harshman & Prout, 1994; Neubaum & Wolfner, 1999; Prout, 1996; Qazi, 2003), although in several of these studies the exact identity of the corresponding SFPs is still unknown. The fact that we did not see a corresponding negative effect of Mlig‐pro46 and Mlig‐pro54 knockdown on *P*_1_ could suggest that it is more likely that they affect displacement of rival sperm rather than affecting own sperm storage or viability. But this missing effect on *P*_1_ could also stem from our experimental design, representing a highly controlled but potentially not very naturalistic mating pattern involving a long series of matings with one partner followed soon after by a long series with a second partner, because there is the possibility that a worm is removing its own sperm during the multiple matings rather than that of rival individuals, so we should be cautious in concluding anything about mechanisms at this stage. Nevertheless, it is interesting in our context that Mlig‐pro46 exhibits a significant homology to the human gene WSCD2 (as do several other *Macrostomum* SFP candidates; Ramm et al., 2019), because expression of WSCD2 is enriched in human male accessory reproductive glands (seminal vesicle, prostate; Uhlén et al., 2015; Uhlen et al., 2010). The other transcripts Mlig‐pro54 (and Mlig‐pro5, see below) are currently unannotated (Grudniewska et al., 2016; Wudarski et al., 2017).

Even if the precise mechanism remains unclear, a reduction in sperm competitive ability upon losing a functionally relevant SFP—as seen for Mlig‐pro46 and Mlig‐pro54—appears straightforward to interpret. That the loss of another transcript—Mlig‐pro5—actually increased sperm competitive ability is harder to explain. One clear possibility is that this again represents an artifact of the experimental design. If, for example, Mlig‐pro5 normally functions to improve sperm displacement but works most effectively on recently deposited sperm, this might in our assay disproportionately have impacted negatively on own sperm from previous matings in the other treatments (depressing *P*_2_) but boosted storage of own sperm in the Mlig‐pro5 knockdown (enhancing *P*_2_). A very similar but converse result, where an individual apparently benefits from the loss of a specific SFP through enhanced defense ability, was reported in *D. melanogaster* following the deletion of Acp62F (Mueller et al., 2008). Another explanation for the increase could be that Mlig‐pro5 acts as a trigger to respond to additional incoming sperm if an initial insemination has already happened, by, for example, decreasing the remating rate or increasing the rate of the suck behavior.

Similar to Mlig‐pro5 in the *P*_2_ assay, where the loss of the SFP resulted in an increased sperm competitive ability, the loss of five individual transcripts showed also a beneficial effect, at least from the donors' perspective, in the fertility assay. The loss of each of the five individual transcripts resulted in an increase in total offspring production. This is somewhat surprising, given that we would normally expect a donor should benefit from stimulating the fecundity of their partner, in a similar way as in *Drosophila* where the SFP ovulin increases egg laying postmating (Herndon & Wolfner, 1995). But like mentioned above, SFPs could also act as a trigger to respond to additional incoming sperm if an initial insemination has already happened, and the loss of this trigger could have a similar effect on the remating rate and the rate of the suck behavior as we suggested for Mlig‐pro5.

Overall, a clear pattern to emerge from our study is that only a minority of knockdowns appear to affect sperm competitive ability and offspring production. One important reason for this is likely to be that several different SFPs can affect the same trait. Such functional redundancy means that even if an individual is lacking one specific SFP, one or more other SFPs still present in the ejaculate compensate for this loss. There are several reported cases of potential functional redundancy among SFPs. In *D. melanogaster*, for example, it is suggested that SFPs other than Acp70A, which has an effect on female receptivity, also affect this trait (Chapman et al., 2003). Acp70A also has a near functionally silent homologue, Dup99B, and the injection of Dup99B mimics the effects of Acp70A (Saudan et al., 2002). There are also at least three antibacterial peptides (Lung, Kuo, & Wolfner, 2001), eight putative proteases, and nine putative protease inhibitors (Swanson et al., 2001) that show potential functional redundancy, but which is interestingly not necessarily explained by sequence similarity. Furthermore, there was functional redundancy demonstrated for ovulin (Herndon & Wolfner, 1995), a SFPs which increase egg laying postmating and also for sex peptide (Chapman et al., 2003; Liu & Kubli, 2003), which induces several postmating responses in the female. Removal of either one does not completely abolish the postmating responses in the female. Additionally, there is also redundancy in tissue targeting of SFPs: More than one SFP targets to any given tissue in the female reproductive tract (Ram, Ji, & Wolfner, 2005).

Another reason for the paucity of detectable impacts of SFP knockdown on reproduction could be that our experimental design integrates over several but certainly not all aspects of sperm competition and would mainly measure immediate impact on sperm competition ability. This potentially misses other targets of SFPs that result in long‐term effects such as manipulation of subsequent behavior (e.g., with respect to remating) or sex allocation (see below). In *Drosophila*, sex peptide is known to be crucial for the long‐term postmating response. Females mated to sex peptide null males remain highly receptive to remating (Chapman et al., 2003; Liu & Kubli, 2003). Because in our *P*_1_ assay remating with the competitor occurred immediately after the first mating, any possible long‐term effect of suppressed receptivity mediated by SFP receipt might have had no impact. In a study where 25 SFPs knockdowns in *Drosophila* were tested for their impact on remating, none of them appeared to modulate the receptivity of the mated female at 24 hr postmating (Ram & Wolfner, 2007b). Females mated to either control or knockdown males showed equally low receptivity to remating. But these authors could identify three SFPs which showed an effect on long‐term receptivity of females: at 4 days postmating, mates of these three knockdown males were significantly more receptive to remating than mates of control males.

Another important target of SFPs in *M. lignano*, which is a simultaneous hermaphrodite, could be the male sex function of the partner which received the ejaculate. *M. lignano* individuals can plastically allocate their resources toward the male or the female sex function (Janicke et al., 2013; Janicke & Schärer, 2010; Schärer & Ladurner, 2003), and donors might conceivably benefit from manipulating this. In the simultaneously hermaphroditic great pond snail *Lymnaea stagnalis*, effects of SFPs on both the male and female functions have been observed. More specifically, the intravaginal injection of one SFP (LyAcp10) affected egg laying (Koene et al., 2010) and the injection of either of two other SFPs (LyAcp8b and LyAcp5) resulted in a reduction of sperm transferred in a subsequent mating by the recipient in *L. stagnalis*, and as a result, in a decrease in their paternity success in subsequent matings as a male (Nakadera et al., 2014; see also Schärer, 2014). This study highlights that steering your partner away from its male function is a potentially adaptive strategy in simultaneous hermaphrodites (Charnov, 1979; Schärer & Ramm, 2016), triggered by seminal fluid.

The relatively small number of functionally relevant SFPs identified in our screen suggests that a more targeted or specific approach is needed to identify more SFP‐mediated effects. Because *M. lignano* is transparent, it is, for example, possible to measure intermediate aspects of sperm competitive ability such as sperm transfer and storage success in vivo (see, e.g., Marie‐Orleach et al., 2016). By measuring the sperm transfer success and the still available sperm number after a certain time or under different scenarios implying more or less competition, we could potentially gain a more precise picture of SFP‐mediated effects on sperm storage and sperm removal. Additionally, it would be beneficial to measure the impact of SFPs on behavioral aspects like the remating rate or the suck behavior described above. And another important aspect, which was not measured with our assay, is the potential influence on the partners' male function, as just discussed. To solve the problem of functional redundancy, it will be necessary to identify proteins which are functionally overlapping or to find ways to transfer specific components or just a small subset of the seminal fluid to a partner. And given that the timing of the mating in our assay might well have affected the outcomes, varying both the pairing time and gap between pairings might well shed new light on underlying sperm competition mechanisms in future studies, and help to disentangle cases where effects of SFPs presumably intended for own sperm actually also impact on rival ejaculates, and vice versa (see, e.g., Nguyen & Moehring, 2018).

Finally, we note that our finding of a mean *P*_2_ of 0.66 is very similar to a previous estimate in *M. lignano* of 0.64 (Sandner, 2013), and such a last “male” sperm precedence fits with previous evidence for sperm displacement in this species (Marie‐Orleach et al., 2014). We know from other taxa that own, competitor, and female genotypes can influence sperm competition outcomes (e.g., Bjork, Starmer, Higginson, Rhodes, & Pitnick, 2007; Clark & Begun, 1998; Clark et al., 1999). Because the three competitor worms for each focal donor in our assay were randomly sampled from an outbred culture, the average *P*_1_ and *P*_2_ scores of the focal donors (as well as those minus specific seminal fluid components) in our assays should therefore be considered as this genotype's general sperm competitive ability against rivals, at least in this recipient (female) genetic background.

In conclusion, we report some evidence for seminal fluid‐mediated fitness effects in the simultaneous hermaphrodite *M. lignano*. Further and more detailed research is now needed to pinpoint precisely how seminal fluid affects sperm competitive ability and fertility, and this transparent flatworm represents a promising model system in which to perform such studies. Additionally, we could show evidence for a potential functional redundancy of SFPs and evidence for SFPs acting as triggers for postmating responses in the sperm recipient.

## CONFLICT OF INTEREST

None declared.

## AUTHOR CONTRIBUTIONS

MW and SAR conceived the study. MW performed the RNAi knockdown and MW and AG the mating assays. MW analyzed results and drafted the manuscript together with SAR. All authors contributed to manuscript revisions and approved the final manuscript.

## Data Availability

Data are available via the Dryad Digital Repository https://doi.org/10.5061/dryad.8w9ghx3gv.
