# Supplementary material for: Seminal fluid‐mediated fitness effects in the simultaneously hermaphroditic flatworm Macrostomum lignano
Source: Ecol Evol. 2019 Nov 18;9(24):13889–901. doi: 10.1002/ece3.5825 (PMC6953679; doi:10.1002/ece3.5825)
Supplement: Supplementary file 3 [file ECE3-9-13889-s003.docx]

**Fig. S1—Representative whole-mount in situ hybridization expression patterns for knockdown of the 18 different seminal fluid transcripts.** ISH was performed for samples of control and knockdown individuals emerged from the experimental assays. ISH patterns for control individuals (left; green) compared to knockdown individuals (right; blue) for the respective transcript.

**Table S1—List of primers used for generating dsRNA for RNAi.** List of Forward and Reverse primers used for generating the dsRNA for the RNAi knock-down for each transcript. Additional to the transcript names we also named the respective transcript IDs from the *de novo* MLRNA110815 transcriptome assembly (Grudniewska et al. 2016), from which the seminal fluid transcripts were identified, as well as the corresponding transcripts in the more recent genome-guided transcriptome assembly Mlig_RNA_3_7_DV1_v3 (Wudarski et al. 2017; Grudniewska et al. 2018).
